# Assessment of the Relationship between Pre-Existing Muscle Atrophy, Subcutaneous Fat Volume, and the Prognosis of COVID-19

**DOI:** 10.3390/jcm14041154

**Published:** 2025-02-11

**Authors:** Fariba Zarei, Afrooz Sepahdar, Mahdi Saeedi-Moghadam, Banafsheh Zeinali-Rafsanjani

**Affiliations:** 1 Medical Imaging Research Center, Shiraz University of Medical Sciences, Shiraz 7134814336, Iran; zareifari@gmail.com; 2 Department of Radiology, Shiraz University of Medical Sciences, Shiraz 7134845794, Iran

**Keywords:** muscle mass, fat, prognosis, COVID-19, obesity

## Abstract

**Background:** Coronavirus disease 2019 (COVID-19), with its rapid transmission and emergence, has become a major global public health concern. COVID-19 patients are at an increased risk of acute skeletal muscle loss and complications such as muscular weakness, depression, and anxiety. Furthermore, sarcopenia has been linked to COVID-19 vulnerability, hospitalization rates, and severity. This study aims to investigate the relationship between pre-existing sarcopenia, subcutaneous fat, and the prognosis of COVID-19. **Methods:** Patients over 18 with positive tests who had chest CT scans without underlying conditions were included in this cohort study. The ratio of muscle volume to the total body surface area was calculated. Patients were divided into four subgroups: outpatients, hospitalized, ICU admitted, and deceased. The initial muscle volume for each group was compared. **Results:** In total, 127 patients were included in the study, 63 (49%) of whom were male. The mean age of the patients was 51.8 ± 18.16 (from 23 to 87 years). In total, 27 patients (21.3%) were treated as outpatients, 49 patients (38.6%) were hospitalized, and 23 (18.1%) were admitted to the ICU. Twenty-eight patients (22%) died. The total muscle/surface area ratio was significantly associated with disease outcome and prognosis. The ratio was significantly lower in dead individuals (*p* = 0.017). **Conclusions:** Sarcopenia was discovered to be significantly associated with the severity of COVID-19 and a poor prognosis. Reducing the risk of severe COVID-19 is possible by identifying and managing sarcopenia-related risk factors.

## 1. Introduction

A potentially fatal respiratory disease known as severe acute respiratory syndrome (SARS) resulted from the SARS coronavirus (SARS-CoV). More recently, SARS-CoV-2, also known as coronavirus 2, has emerged as the seventh human coronavirus to be discovered [1]. Infection with SARS-CoV-2 may result in a broad spectrum of clinical presentations, from mild to severe forms. While a considerable portion of the infected patients are asymptomatic, increased body temperature, shortness of breath, and persistent coughing are common symptoms of COVID-19. Atypical manifestations can be partially related to the disease [2,3,4].

COVID-19 has several complications, including secondary infections and neurological and cardiovascular complications [5,6]. The disease could especially affect muscle health and body composition. Sarcopenia is an age-related condition characterized by progressive loss of muscle mass, strength, and functional capacity and has been pinpointed as one of the important risk factors for serious clinical COVID-19 outcomes [7,8]. The occurrence of age-related muscle decline has been associated with various health complications, such as the development of type 2 diabetes [9], cardiovascular disease [10], frailty, increased fall risk [11], cognitive decline, and depression [12]. These negative effects eventually lead to a lower quality of life and higher mortality rates [13,14]. 

The prevalence of sarcopenia will clearly increase with the aging of the global population, and the number of older adults in need of extended rehabilitation is probably going to be fourfold [15]. This rise can be attributed primarily to this population’s increasing prevalence of musculoskeletal disorders, visual and auditory impairments, fatigue, cognitive decline, sleep disorders, and depression [16,17]. As a result of these factors, sarcopenia increases the susceptibility of older people to COVID-19, and it has been linked to disease severity, resulting in polypharmacy, multi-organ failure, admission to the intensive care unit (ICU), the need for mechanical ventilation, and an increased mortality risk [18,19]. 

The rationale for focusing on muscle atrophy and subcutaneous fat in the context of COVID-19 prognosis is based on their potential physiological relevance. Muscle mass is closely linked to respiratory function and immune response, both of which are critical in the context of a respiratory illness like COVID-19. Sarcopenia, or age-related muscle loss, has been associated with increased susceptibility to severe COVID-19 outcomes, potentially due to weaker respiratory muscles and impaired immune defenses [18,19]. Additionally, excess subcutaneous fat has been linked to heightened inflammation and metabolic dysregulation, which may exacerbate the inflammatory and metabolic derangements observed in severe cases of COVID-19 [20]. Furthermore, the presence of both sarcopenia and obesity, known as sarcopenic obesity, has been identified as a particularly high-risk phenotype, as the combination of muscle loss and central adiposity can lead to compounded metabolic and cardiovascular complications [20]. 

Given these potential mechanistic links, a detailed assessment of muscle and fat distribution in COVID-19 patients may provide valuable insight into the role of body composition in disease severity and prognosis. This study, in particular, will determine the different relationships between muscle mass, subcutaneous fat volume, and the clinical outcome of COVID-19 patients. We also hope to learn more about their roles in influencing disease severity and prognosis through such assessments of body composition variables.

Muscle atrophy in sarcopenic patients has the potential to stimulate the synthesis of myokines, such as interleukin-6 (IL-6) and IL-7, which can have a significant impact on the immune response [21,22]. Furthermore, people with severe or fatal COVID-19 cases have lower protein levels in their bodies. Blood urea nitrogen levels are elevated and serum total protein and albumin levels decrease [22]. Weight loss, reported in many COVID-19 patients, has a heightened manifestation among those requiring ICU treatment [23]. Individuals with severe cases frequently experience fatigue, myalgia, and elevated muscle damage markers such as creatine kinase [22]. This could imply that the most severe respiratory symptoms of COVID-19 occur in patients with the lowest muscle mass, owing to weakened respiratory muscles that probably worsen respiration [24].

Given the potential complexities of muscle analysis in hospitalized COVID-19 patients, particularly in older populations, it is critical to screen these patients for muscle-wasting conditions, such as sarcopenia and cachexia [20]. The clinical situation of older people with obesity and significant muscle loss, which is frequently masked by adipose tissue, poses a complex challenge. The presence of both sarcopenia and obesity, known as sarcopenic obesity, is linked to an increased risk of metabolic and cardiovascular complications [20].

There is a correlation between obesity, specifically central obesity, and an elevated susceptibility to more severe cases of COVID-19 infection [25]. Furthermore, patients with an elevated body mass index (BMI) are more vulnerable to severe disease manifestations. Obesity prevalence is expected to rise steadily until 2030, and it is worth noting that extreme obesity is associated with higher overall mortality rates [26]. Using CT in conjunction with non-invasive post-processing techniques provides an effective method for differentiating and quantifying various adipose tissues, allowing for a more thorough assessment of body fat distribution [27].

The relationship between fat and muscle distribution and the severity of the disease needs more investigation. The objective of this study was to assess the relationship between muscle and subcutaneous fat volumes and the prognosis of COVID-19 patients to explore how these body composition factors might influence the severity and outcome of the disease.

## 2. Materials and Methods

### 2.1. Study Design and Patient Selection

This study enrolled 1200 adults with COVID-19 (18 years and older) who tested positive for PCR between 20 April 2020 and 21 September 2020. In total, 127 eligible patients who had undergone chest CT scans for the first time after the onset of the disease at university-affiliated hospitals and had CT scans with no confounding background diseases were included in the study. The inclusion of patients without underlying medical conditions allowed for the examination of the direct effects of COVID-19 infection on body composition without the potential confounding influence of pre-existing comorbidities. This approach was chosen to provide insights into the specific metabolic and body composition changes associated with the acute phase of COVID-19 disease. Those who had not undergone CT scans for the first time or whose images did not include the T12 level were excluded.

The CT scanners of Philips (Ingenuity, Philips Healthcare, Best, The Netherland), Philips (Brilliance 16, Philips Healthcare, Best, The Netherland), GE (BrightSpeed, GE Healthcare, Waukesha, WI, USA) and GE (LightSpeed, GE Healthcare, Waukesha, WI, USA) were used to perform chest CT scans. The protocol using Philips (Ingenuity) was kV of 120, mAs of 200 ± 50.2, matrix size of 512 × 512, and slice thickness of 4. The protocol using Philips (Brilliance 16) was kV of 120, mAs of 120 ± 25.6, matrix size of 512 × 512, and slice thickness of 2. The protocol using GE (BrightSpeed) was kV of 100, mAs of 40, matrix size of 512 × 512, and slice thickness of 2.5. The protocol using GE (LightSpeed) was kV of 120, mAs of 40, matrix size of 512 × 512, and slice thickness of 1.25.

**Figure 1:**
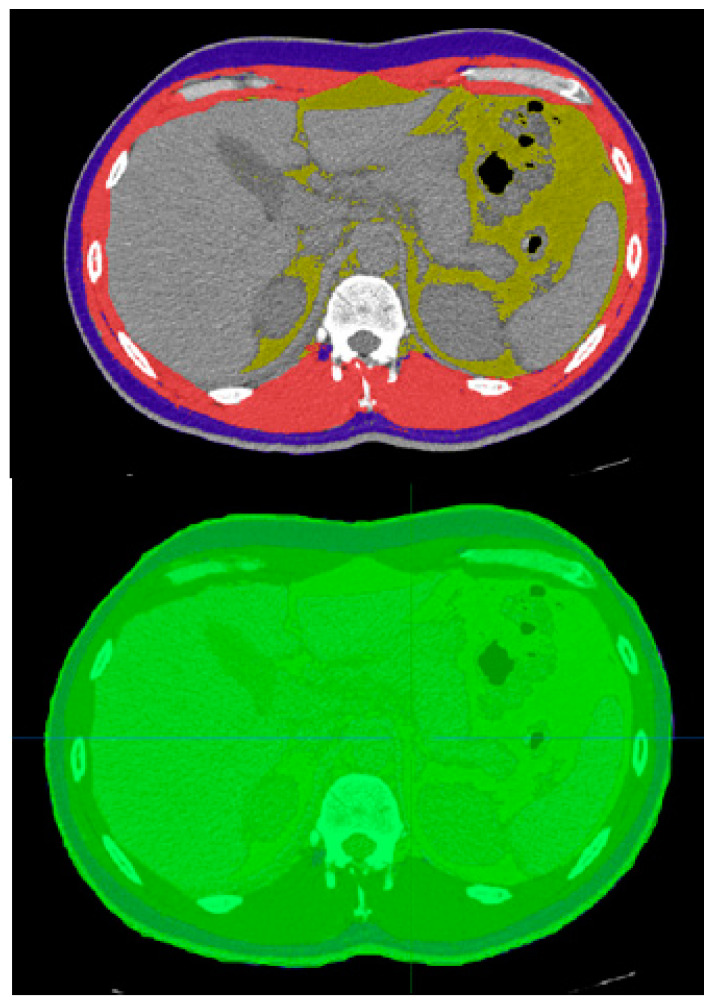
The red, blue, and yellow areas (**upper** image) are total muscles, subcutaneous fat, and visceral fat, respectively, in this section. The total body volume in the mentioned CT cut (i.e., the volume of total body cross-section in this slice (**lower** image, green area)) was measured, then the muscle and fat volume ratio to total body volume was calculated to cancel out the effect of any possible zoom-out or magnification on the image.

Online CoreSlicer software (available at https://old.coreslicer.com/, accessed on 24 Agust 2024) was used to measure muscle and fat volume at the level of the last vertebra to which the rib was attached (Figure 1). Figure 1 also shows visceral fat. However, as chest CT is not suitable for visceral fat measurement, it was not evaluated. The total body volume in the mentioned CT cut was measured, then the muscle and fat volume ratio to total body volume (i.e., the volume of total body cross-section in this slice) was calculated to cancel out the effect of any possible zoom-out or magnification on the image. Muscle/body and subcutaneous fat/body ratios were calculated for each patient and presented as the percentage ratio of volume/body. The volume of the total body cross-section in the slice was measured using the medical interaction toolkit version 2022 (available at https://www.mitk.org/wiki/The_Medical_Imaging_Interaction_Toolkit_(MITK)) via the region growing method. Figure 1 shows the measured volumes.

Patient treatment and follow-ups were assessed using the hospital information system to determine the outcome of their treatment. Accordingly, they were divided into four subgroups: (1) patients managed on an outpatient basis; (2) patients admitted to the general ward; (3) patients admitted to the ICU, intubated, and placed on mechanical ventilation; and (4) deceased patients. These four groups were compared with regard to the initial percentage ratio of muscle and subcutaneous fat volumes in the body. Other variables included age, gender, and treatment outcome.

### 2.2. Data Analysis

The data were inputted into SPSS version 26 for analysis. The mean (standard deviation) was used to express the quantitative data, while the frequency (percentage) was used to present the qualitative data. To assess the differences in means among the various study groups containing non-paired data, first the normality was evaluated by the Shapiro–Wilk test, then for normally distributed data, the ANOVA, or for non-normally distributed data, the non-parametric Mann–Whitney test was employed. The Pearson correlation coefficient was used to evaluate the association between the fat and muscle volumes and the status of the patients. A 95% confidence interval and a two-tailed *p*-value less than 0.05 were considered statistically significant. Unique identifiers were used to code data extracted from medical records, and patient names were not included to protect patient data. Shiraz University of Medical Sciences Ethics Committee approved this study (Ethical code: IR.SUMS.MED.REC.1400.293).

## 3. Results

### 3.1. Descriptive Results

Among the 127 study patients, 63 (49%) were male and 64 (51%) were female, with a mean age of 51.8 ± 18.16 years. Out of all the patients, 27 (21.3%) were treated as outpatients, 49 (38.6%) were hospitalized, 23 (18.1%) were admitted to the ICU, and 28 patients (22%) were deceased. Table 1 shows the descriptive information of each subgroup regarding their age group and sex classification. The mean of total muscle/body and subcutaneous (SQ) fat/body were 0.14 ± 0.028 and 0.19 ± 0.095, respectively (Table 2).

### 3.2. Analytical Results

Table 1 shows that although patient age did not have a significant association with the outcome and prognosis of the disease (*p* = 0.42), among the age groups, the highest mortality rate was observed in the age group above 60 years (32.6%), while the lowest was observed in the 40–60 age group (13.6%). The highest frequency of ICU hospitalizations was observed in the 40–60 age group (29.5%). Among patients aged 20–40 years, 27.5% received outpatient treatment. Moreover, a lack of statistically significant disparity was observed between age, gender, and the final disease outcome or prognosis (*p* = 0.42, and *p* = 0.97). There was a significant difference between the ratio of total muscle/body area amongst different age groups (*p* = 0.010), sex (*p* < 0.001), and outcomes (*p* = 0.012). Specifically, this ratio was significantly lower in deceased patients (*p* = 0.01). 

As expected, the ratio of total muscle/body area was lower in patients with severe outcomes (Pearson correlation = −0.202, *p* = 0.023). A comparison of the total muscle/body ratio of patients with different outcomes also showed a significant difference (ANOVA *p* = 0.01). However, there was no significant relationship between the subcutaneous fat ratio and outcomes (*p* = 0.51) (Table 3).

Table 4 compares the percentage ratio of total muscle and subcutaneous fat to the body between females and males, which shows that women have significantly lower muscle mass and higher subcutaneous fat compared to men (*p* < 0.001). These ratios are also compared in different age groups (Table 5). The results revealed that the percentage ratio of total muscle/body significantly decreased (ANOVA *p* = 0.01, Pearson correlation = −0.264 *p* = 0.003). There was no significant difference in the percentage ratio of subcutaneous fat/body in different age groups (*p* = 0.79).

## 4. Discussion

This study aimed to investigate the effects of sarcopenia and subcutaneous fat on the severity and prognosis of COVID-19 patients. In this study, gender had no significant effect on disease severity. Similarly, prognosis did not differ significantly across age groups, although the age group over 60 had the highest mortality rate.

Among the variables measured in this study, the total muscle/body ratio was significantly related to disease prognosis. Pearson correlation analysis demonstrated a significant negative correlation between the total muscle/body ratio and severe COVID-19 outcomes, indicating that a higher muscle/body ratio was associated with a lower risk of severe disease. One-way ANOVA revealed that the outpatient group had a significantly higher muscle/body ratio compared to the ICU and deceased groups. These findings suggest a connection between increased muscle mass and less severe COVID-19 prognosis.

Similar results have been reported in other studies. Molfino et al. conducted a study to evaluate the nutritional status of COVID-19 patients by measuring skeletal muscle area (SMA) and skeletal muscle index (SMI). They reported that all patients admitted to ICUs had sarcopenia. They also discovered that SMA and SMI were linked to disease complications but had no significant relationship with mortality [28]. In contrast, our study discovered a link between sarcopenia and higher mortality rates. According to Choe et al.’s findings in Malaysia, low muscle mass (LM) was associated with an increased mortality risk in COVID-19 patients admitted to the intensive care unit. They discovered that LM increased the death risk by 2.4 times, regardless of age, disease severity, or organ failure. Furthermore, there was a weak correlation between obesity, BMI, and LM in their study. This was due to 40% of overweight or obese patients experiencing LM [29].

Several other studies have found similar results for various diseases. Toledo et al. [30] discovered that a low skeletal muscle index (SMI) strongly predicted mortality in ICU patients. Moisey et al. [31] discovered that higher SMI was associated with lower mortality in elderly ICU patients. Our findings, along with previous research, highlight the importance of assessing muscle mass as a valuable predictor of survival. This assessment could serve as an initial nutritional status evaluation for risk stratification. It has been suggested that proper nutrition and rehabilitation interventions can help improve muscle status, which could lead to a survival advantage [32].

Unlike our findings, some studies have found subcutaneous fat disparities in patients with different outcomes. Despite age, gender, or inflammatory markers, Peterson et al. [33] found that COVID-19 patients with larger subcutaneous fat areas (SFAs) on CT scans were more likely to require ICU care and mechanical ventilation. However, a significant association was not found between COVID-19 severity and BMI. Watanabe et al. [34] found a statistically significant difference in SFAs, albeit with lower statistical power, whereas BMI did not correlate with severe COVID-19 outcomes.

Another study in 2023 investigated the potential causal relationship between COVID-19 and sarcopenia and its associated traits. However, the results suggested that the association between them might be indirect. They highlight the importance of older individuals ensuring adequate nutrition and exercise to directly manage sarcopenia during the COVID-19 pandemic, as a direct causal relationship was not supported by the analysis [35].

Recently, Koehler et al. [36] conducted a study to evaluate the role of CT-based sarcopenia assessment in predicting intensive care hospitalization among patients with SARS-CoV-2 infection. They measured skeletal muscle mass, muscle density, and body adiposity using CT scans at T4 and L3 levels upon admission. They demonstrated that both thoracic and abdominal sarcopenia are independent predictors of an elevated risk of ICU hospitalization among individuals with SARS-CoV-2 infection.

It is imperative to note that the usefulness of BMI in assessing obesity is predicated on a close relationship between anthropometric measures and direct measures of obesity, such as total body fat or visceral and subcutaneous fat [37]. Several studies have shown that age and gender have a significant influence on body fat levels at a given BMI. The percentage of body fat among women is higher than among men, and the percentage of body fat among older people is higher than among younger people at the same BMI [38,39].

Finally, this study discovered a link between sarcopenia and COVID-19 severity and prognosis. Gender, age groups, and subcutaneous fat, on the other hand, had no significant associations with disease severity or prognosis. These findings imply that measuring muscle mass could be a valuable tool in predicting COVID-19 patient outcomes. More research is needed to fully comprehend the complex relationships between body composition, age, and gender in the context of COVID-19 outcomes.

A limitation of this research lies in the utilized exclusion criteria, potentially affecting the applicability of the results. Patients were specifically excluded if they did not have a confirmed diagnosis of COVID-19. Moreover, individuals were excluded if they had not undergone initial CT scans or if their CT images did not cover the T12 level, which is essential for the body composition analysis. While these exclusion criteria were essential for ensuring the accuracy of the body composition assessments, they may have led to the under-representation of certain COVID-19 patients in the study. It is possible that the associations between body composition and COVID-19 outcomes observed in this group may have varied in a more diverse sample that included a wider range of patients.

## 5. Conclusions

The total muscle/body ratio was significantly associated with increased disease severity and a poor prognosis of COVID-19. No statistically significant correlation was found between subcutaneous fat and disease severity. The identification and effective control of factors that contribute to sarcopenia have the potential to reduce susceptibility to severe COVID-19 infection.

## Figures and Tables

**Table 1:** The relationship between age and gender and the outcome of the patients.

	Subgroups	Outpatient	Ward	ICU	Dead	Total	*p*-Value
Variables							
Age (Years)	20–40	11 (27.5)	16 (40)	5 (12.5)	8 (20)	40	0.42
	40–60	9 (20.5)	16 (36.4)	13 (29.5)	6 (13.6)	44	
	≥60	7 (16.3)	17 (39.5)	5 (11.6)	14 (32.6)	43	
Gender	Male	13 (20.6)	24 (38.1)	11 (17.5)	15 (23.8)	63	0.97
	Female	14 (21.9)	25 (39.1)	12 (18.8)	13 (20.3)	64	

**Table 2:** The mean ± standard deviation of variables related to muscle and subcutaneous fat.

	Variables	Mean ± Standard Deviation (Median)	Minimum	Maximum
Quantities				
Total muscle/body %		0.14 ± 0.028 (0.14)	0.075	0.212
SQ fat/body %		0.19 ± 0.095 (0.17)	0.006	0.45

**Table 3:** The relationship between measured variables (mean ± standard deviation (median)) and the outcome of patients. The *p*-values are calculated using the ANOVA test.

	Variables	Outpatient	Ward	ICU	Dead	*p*-Value
Subgroups						
Total muscle/body %		0.02 ± 0.157 (0.16)	0.02 ± 0.139 (0.14)	0.03 ± 0.143 (0.14)	0.02 ± 0.135 (0.14)	0.01
SQ fat/body %		0.09 ± 0.18 (0.17)	0.09 ± 0.199 (0.17)	0.08 ± 0.169 (0.15)	0.10 ± 0.203 (0.2)	0.51

Note: Total muscle/body represents the proportion of total muscle mass to total body weight. SQ fat/body represents the proportion of subcutaneous fat mass to total body weight. The *p*-values indicate the statistical significance of differences in these body composition metrics across the patient subgroups (outpatient, ward, ICU, deceased).

**Table 4:** The relationship between measured variables (mean ± standard deviation) and sex. The *p*-values are calculated using the ANOVA test.

	Variables	Sex	Mean ± SD	*p*
Subgroups				
Total muscle/body %		Female	0.130 ± 0.022	<0.001
		Male	0.157 ± 0.028	
SQ fat %		Female	0.251 ± 0.087	<0.001
		Male	0.131 ± 0.061	

**Table 5:** The relationship between measured variables and age groups. The *p*-values are calculated using the ANOVA test.

	Variables	Mean ± SD	*p*
Subgroups			
SQ fat%	20–40	0.193 ± 0.095	0.79
	40–60	0.197 ± 0.104	
	>60	0.184 ± 0.090	
	Total	0.191 ± 0.096	
Total muscle/body %	20–40	0.152 ± 0.028	0.01
	40–60	0.144 ± 0.027	
	>60	0.134 ± 0.0274	
	Total	0.143 ± 0.028	

Note: SQ fat% represents the proportion of subcutaneous fat mass to total body weight. Total muscle to body% represents the proportion of total muscle mass to total body weight. The *p*-values indicate the statistical significance of differences in these body composition metrics across the age groups.

## Data Availability

The data supporting this study’s findings are available from the corresponding author, B. Z., upon reasonable request.

## References

[1] Zhou P., Yang X.-L., Wang X.-G., Hu B., Zhang L., Zhang W., Si H.R., Zhu Y., Li B., Huang C.L. (2020). A pneumonia outbreak associated with a new coronavirus of probable bat origin. Nature.

[2] Wang C., Horby P.W., Hayden F.G., Gao G.F. (2020). A novel coronavirus outbreak of global health concern. Lancet.

[3] Wang D., Hu B., Hu C., Zhu F., Liu X., Zhang J., Wang B., Xiang H., Cheng Z., Xiong Y. (2020). Clinical characteristics of 138 hospitalized patients with 2019 novel coronavirus–infected pneumonia in Wuhan, China. JAMA.

[4] Estakhr M., Tabrizi R., Ghotbi Z., Shahabi S., Habibzadeh A., Bashi A., Borhani-Haghighi A. (2022). Is facial nerve palsy an early manifestation of COVID-19? A literature review. Am. J. Med. Sci..

[5] Habibzadeh A., Lankarani K.B., Farjam M., Akbari M., Kashani S.M.A., Karimimoghadam Z., Wang K., Imanieh M.H., Tabrizi R., Ahmadizar F. (2022). Prevalence of fungal drug resistance in COVID-19 infection: A global meta-analysis. Curr. Fungal Infect. Rep..

[6] Desai A.D., Lavelle M., Boursiquot B.C., Wan E.Y. (2022). Long-term complications of COVID-19. Am. J. Physiol.-Cell Physiol..

[7] Cruz-Jentoft A.J., Bahat G., Bauer J., Boirie Y., Bruyère O., Cederholm T., Cooper C., Landi F., Rolland Y., Sayer A.A. (2019). Sarcopenia: Revised European consensus on definition and diagnosis. Age Ageing.

[8] Geisler C., Braun W., Pourhassan M., Schweitzer L., Glüer C.-C., Bosy-Westphal A., Müller M.J. (2016). Gender-specific associations in age-related changes in resting energy expenditure (REE) and MRI measured body composition in healthy Caucasians. J. Gerontol. Ser. A Biomed. Sci. Med. Sci..

[9] Mesinovic J., Zengin A., De Courten B., Ebeling P.R., Scott D. (2019). Sarcopenia and type 2 diabetes mellitus: A bidirectional relationship. Diabetes Metab. Syndr. Obes. Targets Ther..

[10] Bahat G., Ilhan B. (2016). Sarcopenia and the cardiometabolic syndrome: A narrative review. Eur. Geriatr. Med..

[11] Schaap L.A., Van Schoor N.M., Lips P., Visser M. (2018). Associations of sarcopenia definitions, and their components, with the incidence of recurrent falling and fractures: The longitudinal aging study Amsterdam. J. Gerontol. Ser. A.

[12] Hayashi T., Umegaki H., Makino T., Cheng X.W., Shimada H., Kuzuya M. (2019). Association between sarcopenia and depressive mood in urban-dwelling older adults: A cross-sectional study. Geriatr. Gerontol. Int..

[13] Tsekoura M., Kastrinis A., Katsoulaki M., Billis E., Gliatis J. (2017). Sarcopenia and its impact on quality of life. GeNeDis 2016: Genetics and Neurodegeneration.

[14] Sipers W.M., de Blois W., Schols J.M., van Loon L.J., Verdijk L.B. (2019). Sarcopenia is related to mortality in the acutely hospitalized geriatric patient. J. Nutr. Health Aging.

[15] Fávaro-Moreira N.C., Krausch-Hofmann S., Matthys C., Vereecken C., Vanhauwaert E., Declercq A., Bekkering G.E., Duyck J. (2016). Risk factors for malnutrition in older adults: A systematic review of the literature based on longitudinal data. Adv. Nutr..

[16] Vatic M., von Haehling S., Ebner N. (2020). Inflammatory biomarkers of frailty. Exp. Gerontol..

[17] Ali A.M., Kunugi H. (2020). Royal jelly as an intelligent anti-aging agent—A focus on cognitive aging and Alzheimer’s disease: A review. Antioxidants.

[18] Ali A.M., Kunugi H. (2021). Approaches to nutritional screening in patients with Coronavirus Disease 2019 (COVID-19). Int. J. Environ. Res. Public Health.

[19] Tehrani S., Killander A., Åstrand P., Jakobsson J., Gille-Johnson P. (2021). Risk factors for death in adult COVID-19 patients: Frailty predicts fatal outcome in older patients. Int. J. Infect. Dis..

[20] Azzolino D., Saporiti E., Proietti M., Cesari M. (2020). Nutritional considerations in frail older patients with COVID-19. J. Nutr. Health Aging.

[21] Sawaya Y., Ishizaka M., Kubo A., Shiba T., Hirose T., Onoda K., Maruyama H., Urano T. (2020). Association between skeletal muscle mass index and lung function/respiratory muscle strength in older adults requiring long-term care or support. J. Phys. Ther. Sci..

[22] Ali A.M., Kunugi H. (2021). Skeletal muscle damage in COVID-19: A call for action. Medicina.

[23] Haraj N.E., El Aziz S., Chadli A., Dafir A., Mjabber A., Aissaoui O., Barrou L., El Hamidi C.E.K., Nsiri A., Harrar R.A. (2021). Nutritional status assessment in patients with COVID-19 after discharge from the intensive care unit. Clin. Nutr. ESPEN.

[24] Gualtieri P., Falcone C., Romano L., Macheda S., Correale P., Arciello P., Polimeni N., De Lorenzo A. (2020). Body composition findings by computed tomography in SARS-CoV-2 patients: Increased risk of muscle wasting in obesity. Int. J. Mol. Sci..

[25] Pediconi F., Rizzo V., Schiaffino S., Cozzi A., Della Pepa G., Galati F., Catalano C., Sardanelli F. (2021). Visceral adipose tissue area predicts intensive care unit admission in COVID-19 patients. Obes. Res. Clin. Pract..

[26] Abdelaal M., le Roux C.W., Docherty N.G. (2017). Morbidity and mortality associated with obesity. Ann. Transl. Med..

[27] Rosenquist K.J., Pedley A., Massaro J.M., Therkelsen K.E., Murabito J.M., Hoffmann U., Fox C.S. (2013). Visceral and subcutaneous fat quality and cardiometabolic risk. JACC Cardiovasc. Imaging.

[28] Molfino A., Imbimbo G., Rizzo V., Muscaritoli M., Alampi D. (2021). The link between nutritional status and outcomes in COVID-19 patients in ICU: Is obesity or sarcopenia the real problem?. Eur. J. Intern. Med..

[29] Ng C.C., Lee Z.Y., Chan W.Y., Jamaluddin M.F., Tan L.J., Sitaram P.N., Ruslan S.R., Hasan M.S. (2020). Low muscularity as assessed by abdominal computed tomography on intensive care unit admission is associated with mortality in a critically ill Asian population. J. Parenter. Enter. Nutr..

[30] Toledo D.O., Carvalho A.M., Oliveira A.M., Toloi J.M., Silva A.C., de Mattos Farah J.F., Prado C.M., Silva J.M. (2018). The use of computed tomography images as a prognostic marker in critically ill cancer patients. Clin. Nutr. ESPEN.

[31] Moisey L.L., Mourtzakis M., Cotton B.A., Premji T., Heyland D.K., Wade C.E., Bulger E., Kozar R.A. (2013). Skeletal muscle predicts ventilator-free days, ICU-free days, and mortality in elderly ICU patients. Crit. Care.

[32] Heyland D.K., Stapleton R.D., Mourtzakis M., Hough C.L., Morris P., Deutz N.E., Colantuoni E., Day A., Prado C.M., Needham D.M. (2016). Combining nutrition and exercise to optimize survival and recovery from critical illness: Conceptual and methodological issues. Clin. Nutr..

[33] Petersen A., Bressem K., Albrecht J., Thieß H.-M., Vahldiek J., Hamm B., Makowski M.R., Niehues A., Niehues S.M., Adams L.C. (2020). The role of visceral adiposity in the severity of COVID-19: Highlights from a unicenter cross-sectional pilot study in Germany. Metabolism.

[34] Watanabe M., Caruso D., Tuccinardi D., Risi R., Zerunian M., Polici M., Pucciarelli F., Tarallo M., Strigari L., Manfrini S. (2020). Visceral fat shows the strongest association with the need of intensive care in patients with COVID-19. Metabolism.

[35] Liu C., Liu N., Zeng Y., Xiao B., Wang P., Zhou C., Xia Y., Zhao Z., Xiao T., Li H. (2023). COVID-19 and sarcopenia-related traits: A bidirectional Mendelian randomization study. Front. Endocrinol..

[36] Koehler J., Boirie Y., Bensid L., Pereira B., Ghelis N., Dupuis C., Tournadre A., Boyer L., Cassagnes L. (2022). Thoracic sarcopenia as a predictive factor of SARS-CoV-2 evolution. Clin. Nutr..

[37] Simonnet A., Chetboun M., Poissy J., Raverdy V., Noulette J., Duhamel A., Labreuche J., Mathieu D., Pattou F., Jourdain M. (2020). High prevalence of obesity in severe acute respiratory syndrome coronavirus-2 (SARS-CoV-2) requiring invasive mechanical ventilation. Obesity.

[38] Demerath E.W., Sun S.S., Rogers N., Lee M., Reed D., Choh A.C., Couch W., Czerwinski S.A., Chumlea W.C., Siervogel R.M. (2007). Anatomical patterning of visceral adipose tissue: Race, sex, and age variation. Obesity.

[39] Després J.-P., Couillard C., Gagnon J., Bergeron J., Leon A.S., Rao D., Skinner J.S., Wilmore J.H., Bouchard C. (2000). Race, visceral adipose tissue, plasma lipids, and lipoprotein lipase activity in men and women: The Health, Risk Factors, Exercise Training, and Genetics (HERITAGE) family study. Arterioscler. Thromb. Vasc. Biol..

